# Percutaneous High Frequency Microwave Ablation of Uterine Fibroids: Systematic Review

**DOI:** 10.1155/2018/2360107

**Published:** 2018-01-08

**Authors:** Anna Maria Ierardi, Valeria Savasi, Salvatore Alessio Angileri, Mario Petrillo, Sara Sbaraini, Antonio Pinto, Francesco Hanozet, Anna Maria Marconi, Gianpaolo Carrafiello

**Affiliations:** ^1^Department of Diagnostic and Interventional Radiology, San Paolo Hospital Medical School, University of Milan, Via A. Di Rudinì 8, 20142 Milan, Italy; ^2^Unit of Obstetrics and Gynecology, Department of Biomedical and Clinical Sciences, ASST Fatebenefratelli Sacco, Hospital “L. Sacco”, University of Milan, 20157 Milan, Italy; ^3^Department of Radiology, Cardarelli Hospital, 80123 Naples, Italy; ^4^Unit of Obstetrics and Gynecology, Department of Health Sciences, San Paolo Hospital Medical School, University of Milan, Via A. di Rudinì 8, 20142 Milan, Italy

## Abstract

Uterine fibroids are the most common benign pelvic tumor of the female genital tract and tend to increase with age; they cause menorrhagia, dysmenorrhea, pelvic pressure symptoms, back pain, and subfertility. Currently, the management is based mainly on medical or surgical approaches. The nonsurgical and minimally invasive therapies are emerging approaches that to the state of the art include uterine artery embolization (UAE), image-guided thermal ablation techniques like magnetic resonance-guided focused ultrasound surgery (MRgFUS) or radiofrequency ablation (RF), and percutaneous microwave ablation (PMWA). The purpose of the present review is to describe feasibility results and safety of PMWA according to largest studies available in current literature. Moreover technical aspects of the procedure were analyzed providing important data on large scale about potential efficacy of PMWA in clinical setting. However larger studies with international registries and randomized, prospective trials are still needed to better demonstrate the expanding benefits of PMWA in the management of uterine fibroids.

## 1. Introduction

Uterine fibroids (leiomyomas) are the most common benign pelvic tumors in reproductive age group women. It occurs in approximately 20–40% of women in this age group and about a quarter among them will have significant clinical symptoms, such as menorrhagia, dysmenorrhea, pelvic pressure symptoms, back pain, subfertility, and reduced quality of life [1–3].

Uterine fibroids are categorized by their location in the uterus as intramural (entirely or mostly contained within the myometrium), submucosal (projecting into the endometrial cavity and may also be pedunculated), or subserosal (projecting outward from the serosal surface of uterus and may be pedunculated). Uterine fibroids are estrogen-dependent and they can enlarge during pregnancy or with the use of oral contraceptive pills and shrink after menopause. Fibroids can be subject to a wide variety of degenerative phenomena, especially during rapid growth including myxoid, hyaline, cystic, red (hemorrhagic), and fatty degeneration as well as calcification and necrosis. These all contribute to the complexity and variability of fibroid imaging appearance.

Currently, the management options included are medical (hormonal and nonhormonal), nonsurgical, and surgical (myomectomy and hysterectomy) [4–6].

The nonsurgical and minimally invasive therapies include uterine artery embolization (UAE) [7, 8] and image-guided thermal ablation techniques: magnetic resonance-guided focused ultrasound surgery (MRgFUS) [9–11], radiofrequency ablation (RF) [12], and percutaneous microwave ablation (PMWA) [13, 14].

According to reported literature, UAE involves occlusion of uterine arteries bilaterally using particulate emboli resulting in ischemic necrosis of the fibroid. This decrease in fibroid volume leads to symptomatic relief. Common concerns with UAE include postprocedural pain, postembolization syndrome, and risk of infection. Rarer complications include premature ovarian failure and endometrial atrophy.

Patient satisfaction rate following UAE when compared with the surgical modality is similar at 2- and 5-year interval. UAE was associated with higher need for surgical intervention (between 15 and 32%) after 2 years [20].

MRgFUS uses a high-intensity ultrasound beam to increase the local temperature of the targeted tissue leading to necrosis and destruction of the tissue. The magnetic resonance is used for planning and controlling the ablative process, restricting it mainly to the selected tissue, thereby avoiding damage to adjacent structures. MRgFUS appears to be safe and an effective modality for treating symptomatic fibroids [9].

In a study that evaluates clinical outcomes after session of MRgFUS for uterine fibroids, a total of 109 results measured by the symptom severity score (SSS) were greater than predicted, with subjects having a mean decrease of 39% and 36% at 6 and 12 months, respectively [9].

Some of the minor complications include skin burns, abdominal wall oedema, and febrile morbidity. Serious complications, such as deep vein thrombosis, bowel injury, persistent neuropathies, and need for emergency hysterectomies, have been reported.

The major limitation of MRgFUS currently is that many women are not eligible for the procedure because of the bowel interposition between ultrasound beam and fibroid or other reasons like more than five fibroids, size or shape of fibroids, or presence of adenomyosis. Furthermore, the treatment of large fibroids with MRgFUS is a long time procedure. Also the cost effectiveness of MRgFUS is still under debate. First-line treatment of eligible women with MRI-guided focused ultrasound is a cost-effective noninvasive strategy. For those not eligible for MRI-guided focused ultrasound, UAE remains a cost-effective option [21].

The radiofrequency ablation was done laparoscopically and found to be safe and efficacious; recently the transvaginal approach has been introduced to deliver the energy [22, 23].

Ablation with microwave has several intrinsic advantages over RFA, including the capability to generate very high tissue temperature, less intraprocedural pain, larger coagulation zones, less sensitivity to tissue type and charring, improved performance near blood vessels, and no requirement of ground pads [24].

Ultrasound (US) guided percutaneous microwave ablation (PMWA) is minimally invasive, has low time requirements, is easy to perform, and has been broadly used for the treatments of solid tumors in organs other than the uterus with favourable effects.

The purpose of the present review is to describe feasibility and safety of the PMWA of uterine fibroids. Moreover, we analyzed technical aspects of the procedure, results, and effectiveness.

## 2. Materials and Methods

### 2.1. Study Selection

A systematic literature search was performed using the PubMed databases for studies published in the English language from January 2005 to March 2017, with the syntax interventional radiology, percutaneous thermal ablation, percutaneous microwave ablation (or MWA), uterine fibroid, and symptomatic uterine fibroid.

Only articles that described percutaneous ablation with microwave in uterine fibroids were included.

We identified additional studies through manual search of the primary studies references, review articles, and key journals.

We excluded papers that included data reported previously.

The following variables were extracted, where available, from the included articles: number of patients; dimension of the fibroids treated; technology used; number of antenna placements and total time of ablation, volume of ablation, and sessions of treatments; technical and clinical success; complications; follow-up; second treatment; surgery (yes or not).

The primary endpoint was to investigate feasibility and safety of the technique. The secondary endpoint was to evaluate effectiveness in terms of improvement of symptoms and quality of life (QOL) and reduction in volume of the uterine myomas.

In Table 1, data extrapolated from the studies analyzed were described.

The feasibility was defined as technical success rate, in particular as the correct positioning of the antennas within the fibroid using sovrapubic US as imaging guidance.

Clinical success was evaluated thorough clinical evaluation, which included the symptoms severity score of the Uterine Fibroids and the Quality of Life questionnaire [25].

All papers reported complications; they were classified according to the Common Terminology Criteria for Adverse Events (CTCAE) classification [26].

Safety was evaluated on the basis of the complications that were recorded immediately after the procedure and during the follow-up [27]. A complication was defined as “immediate” when it occurred up to 6–24 h following the procedure, as “periprocedural” if it occurred within 30 days, and as “delayed” if it occurred more than 30 days after the procedure [27]. Major complications were defined as complications that, if untreated, might threaten the patient's life, lead to substantial morbidity and disability, result in hospital admission, or substantially lengthen the patient's hospital stay [26, 28]. Minor complications included typical postablation syndrome symptoms (fever, pain, nausea, and vomiting) if present > 4 days after the ablation procedure. Complications were further divided into two causal categories: those secondary to the MW antenna placement (bowel perforation, infection, and bleeding) and those secondary to thermal injury (damage to adjacent organs) [26].

### 2.2. Technical Aspects

During PWMA, the antennas are accurately inserted into the fibroid using ultrasound transabdominal guidance, and the microwave generator located in the front of the ablation electrode emits an electromagnetic wave with a frequency of 2450 MHz, as indicated in all studies [13, 15–18] except Liu et al. [19]. The power ranged from 50 W to 100 W in the studies reported, when the information is available.

When reported (Table 1) one antenna was used if the maximum diameter of the fibroid is less than 5 cm; otherwise 2 antennas were deployed. Not all authors reported ablation time.

### 2.3. Pretreatment and Clinical Aspects

**Figure 1:**
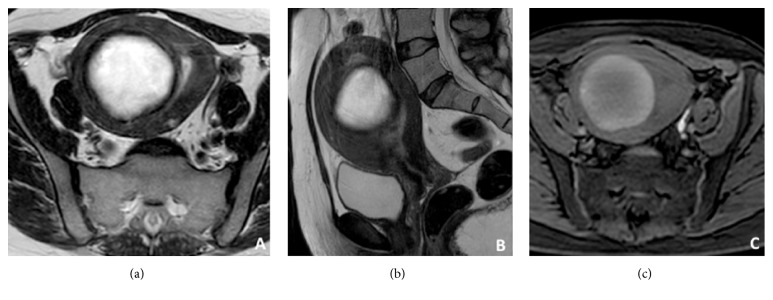
T2w MRI axial (a) and sagittal (b) images show an intramural myoma; T1W-FS MR axial image confirmed a well capsulated centrally hyalinized uterine lesion (c).

In all patients [13, 15–19] uterine fibroids have been diagnosed using ultrasonography (transvaginal and/or sovrapubic) and contrast-enhanced MRI (CE-MRI) (Figures 1(a), 1(b), and 1(c)); in some studies before treatment biopsy was performed [13].

The aim of the pretreatment imaging evaluation is to determine the number, dimensions, and location of the myomas.

In all studies [13, 15–19, 29] the mean diameter and volume of the fibroids were calculated according to retrospective formulas (length + width + height)/3 and 4/3*π*(*d*/2)^3^ before and after the ablation via CEUS (contrast-enhanced ultrasonography) or MRI.

In all studies [13, 15–19] indications or inclusion criteria are similar. In particular, uterine fibroids are symptomatic in all cases (pain, menorrhagia, anemia, and urinary frequency); they are not responsive to medication or other conservative treatment, and women present a strong desire to preserve uterus, and they do not use hormonal drugs usually within the first 3 months of ablation; exclusion criteria comprise desire of future pregnancy, malignant neoplasms of any organ; acute pelvic inflammation; and severe coagulation disorder.

In the studies reporting symptomatic aspects (Table 1) patients underwent a thorough clinical evaluation, which included the symptom severity score of the Uterine Fibroids Symptom and Quality of Life questionnaire [25]. The questionnaire consists of eight questions addressing the frequency and severity of symptoms and 29 questions on health-related quality of life (QOL). Two distinct scores were calculated for symptom severity and QOL. Higher symptom scores are indicative of greater symptom severity, and higher QOL scores indicate a better health-related QOL.

### 2.4. Posttreatment Evaluation

The evaluation of diameter and volume reduction and the changes in symptom score and in health-related QOL represent the criteria reported to evaluate effectiveness of the procedure. Clinical evaluation was performed every 3 months after treatment.

Moreover in some studies, clinical success in terms of severity of symptoms was based on hemoglobin (Hb) levels measured before and after ablation procedures and after every 3 months (Table 1).

**Figure 2:**
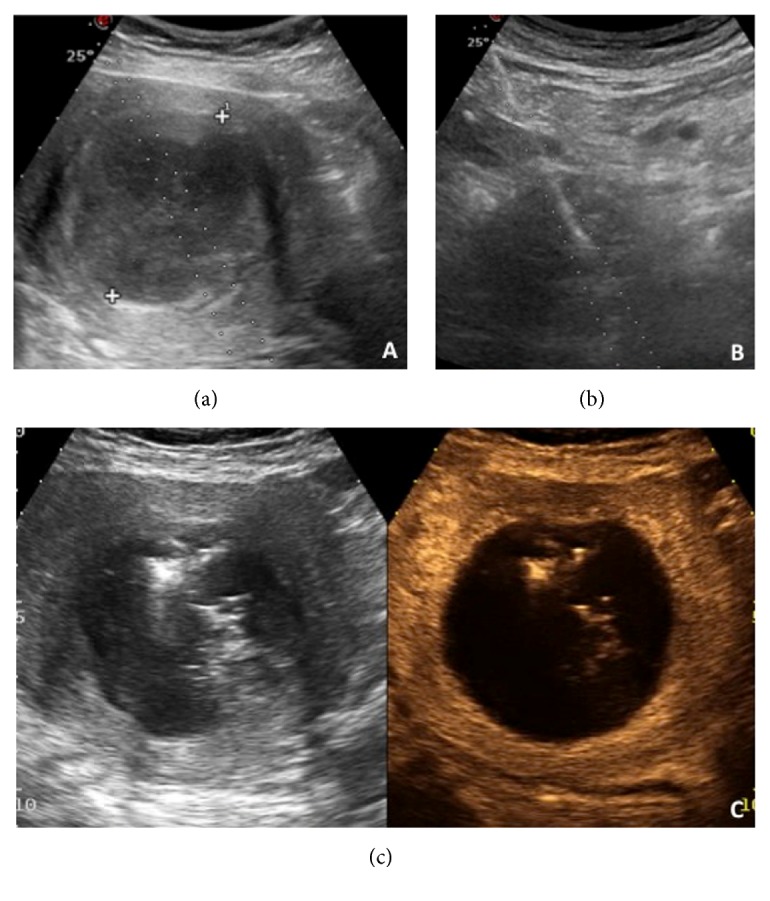
B-mode sovrapubic ultrasound image shows the myoma and the planned path for the insertion of antenna (a); B-mode sovrapubic ultrasound image shows the antenna correctly positioned within the myoma (b); CEUS performed the day after the procedure reveals the desired volume of ablation within the myoma (c).

On the basis of available data (Table 1), contrast-enhanced US (CEUS) is usually performed immediately after the procedure and/or the day after to evaluate the volume of ablation (Figures 2(a), 2(b), and 2(c)). CE-MRI and/or CEUS are used during follow-up to evaluate the shrinkage of the lesion.

## 3. Results

All the results are summarized in Table 1.

In a total of six articles [13, 15–19] the overall experience of percutaneous microwave ablation of fibroids was reported. A total of 541 patients with 647 fibroids were treated. The study with the highest number of treated fibroids was recently published by Liu et al. (311 patients and 405 leiomyomas) with a size ranging between 2.6 and 10 centimeters.

As reported above, a single antenna was usually used for ablation; in large fibroids (>5 cm) authors decided to use double antenna; this decision depends also on different MWA generators available. All generators considered were based on a 2450 Mhz ablation frequency with a power ranging from 50 to 100 W. In particular, according to power and ablation expected volumes, one antenna was applied for fibroids with a diameter < 5 cm and double antennas were applied for fibroids with diameter > 5 cm [18], with ablation time ranging between 300 and 600 seconds. Zhang et al. [13, 15] used single antenna and an ablation time of 300 s and 490 s, respectively. The gauge diameter of each antenna ranged between 15 and 16 based on available equipment, with length ranging between 18 cm and 20 cm (Table 1). Zhang et al. [13] used to fill the bladder with saline prior each ablation, to optimize fibroid position and put them near abdominal wall obtaining at the same time a better visualization of uterus and bladder itself in order to reduce possible damage during ablation procedure. In all considered studies (Table 1) a technical success of 100% was reported with a whole ablated volume percentage equal to 100% for fibroids that were <5 cm in diameter, when ablated with a single antenna.

Clinical success in terms of volume reduction rate was from 15,9% to 93.1% (Table 1). This wide range of variability depends on the different time of instrumental follow-up (immediately after procedure and after 6 months).

In the series analyzed, only Liu et al. [19] reported a twice treatment in 2 patients with more than 10 fibroids to ensure that the ablation was successful with an overall reduction rate in their series of 86.7% at 12 months.

Different results were obtained according different follow-up examinations performed at different time points using Contrast-Enhanced Ultrasound (CEUS) or Contrast-Enhanced Magnetic Resonance Imaging (CE-MRI) with best shrinkage rate of 93.1% (range 61.8–93.1%) reached by Zhang et al. after 12 months [13].

Studies in which clinical success was evaluated in terms of Hb levels measured before and after ablation procedures showed an interesting improvement in Hb level (Table 1) (from 88,64 g/l to 123,21 g/l at 3 months), when considered as a part of clinical success [17, 19].

Clinical success in terms of improvement of the quality of life (QOL) or health-related quality of life (HROL) measured using the Uterine Fibroids Symptom and Quality of Life (UFS-QOL) questionnaire reached normal level at twelfth month according to Yang et al. results [17], or a significant improvement in scores (*p* value < 0.05%) after treatment, according to Liu et al. data [19].

No major complications were observed, as those requiring further interventions and/or hospitalization, according Common Terminology Criteria for Adverse Events (CTCAE) [26].

Minor complications were observed, in particular lower abdominal pain, with a 4/5-point scale value in 7 patients and 2/5 in 3 patients, in Yang et al. series [17]. A frequent reported data was discharge of bloody fluids for no more than 20 days and fragments of necrotic tissues from vagina in multiple cases. These phenomena were judged normally and frequently observed in all series, particularly in those of Zhang et al. [13, 17–19]; they considered quite normal side effects due to endometrial inflammation and irritation caused by necrotizing liquefaction following ablation treatments.

On the basis of the data reported (Table 1), we can hypothesize that a surgical revision (myomectomy or hysterectomy) as a consequence of the ablation procedure or as failure of the percutaneous ablation and consequent persistence of the symptoms was never necessary, but this information is not reported in any study.

## 4. Discussion

Currently, in situ ablation techniques have enabled a great advancement in conservative treatments of uterine myomas [13, 20, 30, 31]. When compared with other thermal ablation techniques, microwaves ablations achieve higher intratumoral temperatures and larger ablations zone [32].

In this review, we found that PMWA of medium-sized and large uterine myomas alone is a feasible and safe procedure that has good short-term follow-up results.

Microwave ablation is less expensive than MRgFUS, easy, and efficient procedure that can be repeated in the same session or at a later time [13]. Microwave generators are widely available in many hospitals for other tumor ablation therapies, and the equipment is simple to use. Larger areas of necrosis (up to 6 cm in diameter) can be achieved in a single access with a single antenna than can be achieved with other thermal ablative tools, particularly laser fibers and monopolar and bipolar needles. The advantage of using a single insertion is reduction of the risk of injury and adhesions [29, 33].

Uterine fibroids are benign lesions, so the main purpose of the treatment is to relieve clinical symptoms and improve patient quality of life. Partial ablation in particular in a relatively unsafe position such as close to bowel or bladder is enough to obtain good clinical results. From a technical point of view injection of saline solution among the bowel loops could be useful in some cases [33].

In our institution, we use a single antenna and we withdraw or reinsert it within the myoma for another session of ablation with the only aim to create a volume of ablation contained in the myoma respecting safety margins, as described by Zhang et al. [13]: during the ablation, variations in the echo from the fibroid were monitored by real-time ultrasonography. Moreover, the reduction of the volume of the myomas seems to be related to the sarcoid percentage of the lesion; for example, Liu et al. [19] reported a slightly lower reduction of volume after 12 months related to intramural leiomyomas [18].

The use of CEUS has the advantage of precise targeting within highly vascularized areas. CEUS depicts changes in tissue echotexture during the procedure and the presence of residual viable tissue, which is useful for assessing the success of the procedure. CEUS can be used in follow-up to predict clinical failure or recurrence. In our experience, CEUS has been a readily available and reliable tool for fast evaluation of residual vascularization [29].

Microwave ablation has a low rate of complications, as reported in this review. In our study, we found no major complications, including bleeding. Bleeding can be successfully prevented by the use of a track ablation technique (cauterization of the needle tract at the end of the ablation) to coagulate perimyoma vessels along the access path: we always used this technique and we suppose that is a common technical practice but in the studies reported it is not specified.

None reported data about the risk of adhesions, but we suppose that none is able to evaluate the rate of adhesion because none of the patients needed second-look laparoscopic or abdominal surgery. Adhesions are instead a consolidated risk with laparoscopic and conventional surgical approaches [34].

In literature only a comparative study between PMWA and USgHIFU is present [18]; authors showed that treatment time was shorter in the PMWA group compared to the USgHIFU group (median treatment time was 46.2 min versus 92.5 min, resp.), producing a larger zone of ablation in a shorter amount of time. The primary reason for this difference in results is likely related to the principle mechanism of action of the two therapy methods. The fibroid ablation rate, average regression rate, and drop in SSS at 6 months after treatment were similar between the two groups, respectively USgHIFU (77.1 ± 18.2%, 50.3% and 9.4) and PMWA (79.8 ± 14.9%, 52.4% and 10.2).

Both therapies did not require general anesthesia and usually only one night of hospitalization is required for PMWA.

UAE is performed under conscious sedation, spinal/epidural, and sometimes general anesthesia; common concerns with UAE include postprocedural pain, postembolization syndrome and risk of infection [35]. This procedure is less endured by patients compared with ablative techniques in terms of intra- and postprocedural pain and risk of infections [35].

The effect of this minimally invasive procedures on fertility is debatable [35]: nowadays no established data are available even if Kim et al. [36] reported 3 cases of uncomplicated pregnancies in 69 patients treated with radiofrequency ablation. Ovarian reserve appears to be affected by UAE in premenopausal women [37].

HIFU seems to preserve ovarian reserve more than the above-mentioned procedures but data available are only preliminary [38].

## 5. Conclusions

Currently multiple treatment options are available for uterine fibroids.

The choice of treatment depends largely on a variety of factors related to patient, operator, and center considered.

No randomized studies exist to compare treatments.

In conclusion, the current study demonstrates the feasibility, safety, and potential efficacy of percutaneous microwave ablation of uterine fibroids.

The specific role of PMWA in the management of uterine fibroids may be considered under investigation. Larger studies with the help of international registries and ideally large, randomized, prospective trials are much needed to better demonstrate the benefits of thermal ablation therapies in the management of uterine fibroids and to help operator to choose a technique compared to another.

## Figures and Tables

**Table 1:** The table summarizes all reviewed series according to each variable included in review process. The following are reported specific acronyms: Pts: patients; CE-MRI: Contrast-Enhanced Magnetic Resonance Imaging; CEUS: Contrast-Enhanced Ultrasound; FUP: follow UpNA: not available; Hb: hemoglobin level; SSS: symptom severity scores. Questions regarding the severity of symptoms; scale of score was 5–40; QMAV: Quantitative Microwave Ablation Volume. The nonenhanced CEUS volume after 50 W × 300 sec or 60 W × 300 sec ablation; HRQL: Health Related Quality of Life; UFS-QOL: Uterine Fibroid Symptom and Quality Of Life.

Study	Pts	Dimension fibroids treated	Technology	Number of antennas	Median treatment time	Posttreatment			Technical success	Clinical success	Complications	FUP	Second treatment	Surgery yes/no
						Ablation rate	CE-MRI/CEUS	Timing						
Zhang et al. 2011 [13]	40 fibroids	Volume mean: 140,1 +/− 87,4 cm^3^	KV2000, 2450 MHz, 50 W 300–600 min, antenna 15 G	1 antenna for <5 cm fibroids Double antennas for >5 cm fibroids	490 sec	Shrinkage rate:	CE-MRI		100%	NA	Lower abdominal pain (6 pz) Small amount vaginal bloody secretions (7 pz → 6 of these recovered for 1 week)	12 m	0	NA
						61,8%		3 months						
						78,7%		6 months						
						73,2%		9 months						
						93,1%		12 months						
	

Xia et al. 2014 [15]	88 pts 91 leiomyomas (T2WI signal: 35 hypointense; 29 isointense; 27 hyperintense)	Volume: 158,09 +/− 127,28 cm^3^	KV2000 tumor coagulator Power: 50 W or 60 W Frequency: 2450 MHz Electrode diameter: 15 G	NA	300 sec	QMAV: Hypointense: 46,58 +/− 25,63 cm^3^ Isointense: 44.46 +/− 16.72 cm^3^ Hyperintense: 23,58 +/− 11,85 cm^3^	CEUS	After 50 W × 300 sec or 60 W × 300 sec's ablation	100%	NA	NA	NA	0	NA
							CE-MRI	Within 5 days after treatment						

Xia et al. 2014 [16]	49 pts 49 leiomyomas (T2WI signal: 28 hypointense; 13 Isointense; 8 hyperintense)	Average diameter: intramural/subserous myoma 5 cm; submucous myoma 3 cm	KV2000 tumor coagulator: Power 50 W Frequency: 2450 MHz Electrode diameter: 15 G	1 antenna for <5 cm fibroids Double antennas for >5 cm fibroids	NA	NA	CEUS	At the end of treatment to evaluate ablation effects	100%	NA	NA	NA	0	NA
							CE-MRI	Within 7 days after treatment						

Yang et al. 2014 [17]	22 pts 22 submucosal myomas	Diameter: 4,9 cm	KY2000 MW, 2450 MHz, antenna 15 G	100% single ablation	NA	Volume reduction rate: 81,46% 90%	CE-MRI and CEUS	3 months 12 months	100%	Hb: from 88,64 g/l to 123,21 g/l at 3 months and to 125,92 at 12 months UFS-QOL: normal level at 1 year	No major complications Lower abdominal pain (31,82%) Small amount of vaginal secretion (100%) Bloody vaginal secretion (9%)	12 m	0	NA

Zhao et al. 2015 [18]	31 pts 40 fibroids	Average diameter: 68,6 +/− 12,7 mm	KV2100 Microwave tumor treatment device: Power 50–100 W Frequency: 2450 MHz Electrode diameter: 15 G	1 electrode for <5 cm fibroids Double electrode for >5 cm fibroids	Session 46,2 min; MWA NA	Ablation rate: 79,8 +/− 14,9%	CEUS	Immediately after procedure	100%	Changes in SSS: 10.2	Lower abdomen pain Vaginal discharge Low grade fever NO serious adverse events	6 m	0	NA
						Regression rate: 52,4%	CE-MRI	After 6 months						

Liu et al. 2016 [19]	311 pts 405 leiomyomas	Diameter: 5,1 +/− 1,28 cm Volume: 95,01 +/− 70,29 cm^3^	KV2000 MW + ECO-100C Microwave system	1 or 2 microwave antennas	NA	Reduction rate: 63,5% 78,5% 86,7%	CEUS	3 months 6 months 12 months	100%	Hb: from 88,84 +/− 9,31 g/l to 107,14 +/− 13,32 at 3 months and to 117,79 +/− 6,51 at 12 months SSS and HRQL significantly improve post treatment	Lower abdominal pain (8,68%). Small amount of vaginal secretion (6,11%)	12 m	2 pts	NA
